# Modulating Golgi Stress Signaling Ameliorates Cell Morphological Phenotypes Induced by CHMP2B with Frontotemporal Dementia-Associated p.Asp148Tyr

**DOI:** 10.3390/cimb46020090

**Published:** 2024-02-05

**Authors:** Shoya Fukatsu, Maho Okawa, Miyu Okabe, Mizuka Cho, Mikinori Isogai, Takanori Yokoi, Remina Shirai, Hiroaki Oizumi, Masahiro Yamamoto, Katsuya Ohbuchi, Yuki Miyamoto, Junji Yamauchi

**Affiliations:** 1 Laboratory of Molecular Neurology, Tokyo University of Pharmacy and Life Sciences, Tokyo 192-0392, Japans207076@toyaku.ac.jp (T.Y.); miyamoto-y@ncchd.go.jp (Y.M.); 2 Tsumura Research Laboratories, Tsumura & Co., Inashiki 200-1192, Japan; ooizumi_hiroaki@mail.tsumura.co.jp (H.O.); hirokoma@h.email.ne.jp (M.Y.); oobuchi_katsuya@mail.tsumura.co.jp (K.O.); 3 Laboratory of Molecular Pharmacology, National Research Institute for Child Health and Development, Setagaya, Tokyo 157-8535, Japan; 4 Diabetic Neuropathy Project, Tokyo Metropolitan Institute of Medical Science, Setagaya, Tokyo 156-8506, Japan

**Keywords:** CHMP2B, Golgi stress, Hsp47, Arf4, differentiation

## Abstract

Some charged multivesicular body protein 2B (CHMP2B) mutations are associated with autosomal-dominant neurodegenerative frontotemporal dementia and/or amyotrophic lateral sclerosis type 7 (FTDALS7). The main aim of this study is to clarify the relationship between the expression of mutated CHMP2B protein displaying FTD symptoms and defective neuronal differentiation. First, we illustrate that the expression of CHMP2B with the Asp148Tyr (D148Y) mutation, which preferentially displays FTD phenotypes, blunts neurite process elongation in rat primary cortical neurons. Similar results were observed in the N1E-115 cell line, a model that undergoes neurite elongation. Second, these effects were also accompanied by changes in neuronal differentiation marker protein expression. Third, wild-type CHMP2B protein was indeed localized in the endosomal sorting complexes required to transport (ESCRT)-like structures throughout the cytoplasm. In contrast, CHMP2B with the D148Y mutation exhibited aggregation-like structures and accumulated in the Golgi body. Fourth, among currently known Golgi stress regulators, the expression levels of Hsp47, which has protective effects on the Golgi body, were decreased in cells expressing CHMP2B with the D148Y mutation. Fifth, Arf4, another Golgi stress-signaling molecule, was increased in mutant-expressing cells. Finally, when transfecting Hsp47 or knocking down Arf4 with small interfering (si)RNA, cellular phenotypes in mutant-expressing cells were recovered. These results suggest that CHMP2B with the D148Y mutation, acting through Golgi stress signaling, is negatively involved in the regulation of neuronal cell morphological differentiation, providing evidence that a molecule controlling Golgi stress may be one of the potential FTD therapeutic targets at the molecular and cellular levels.

## 1. Introduction

Frontotemporal dementia (FTD) and/or amyotrophic lateral sclerosis (ALS) are genetically heterogeneous autosomal-dominant neurodegenerative diseases [1,2,3,4,5,6,7,8]. As one example, specific nucleotide repeat expansions in the *c9orf72* gene and mutations in some other genes are well known to be associated with both diseases [1,2,3,4]. At present, FTD and ALS are considered to be a single-spectrum disorder. FTDALS often begins in adulthood, and in some cases, it occurs before the age of 10 years. FTDALS-associated gene products likely converge on the common pathological molecular pathway(s). It is believed that the common disease-related pathway(s) may explain the overlap in clinical symptoms [1,2,3,4,5,6,7,8]. One of the major molecular pathological mechanisms of FTDALS is responsible for the abnormality or deficiency of proteostasis, such as unsuitable timing and an abnormal amount of protein degradation [5,6,7,8,9,10,11,12]. The networks of proteostasis include integrated cellular biological pathways in living cells and control the biosynthesis, folding, transport, and degradation of a variety of proteins on the inside, surface, and outside of cells.

Charged multivesicular body protein 2B (CHMP2B) is one such major molecule composing proteostasis networks and is involved in the regulation of the intracellular biomaterial trafficking coupling the endosome to the lysosome [13,14,15,16]. CHMP2B is a core component of the endosomal sorting complex required for transport (ESCRT) machinery. ESCRT machinery is composed of ESCRT-0, ESCRT-I, ESCRT-II, and ESCRT-III. Importantly, the machinery contributes to remodeling intracellular membranes [17]. By sequentially binding to ubiquitin-conjugated proteins, ubiquitin-conjugated proteins are sequestered within the internal vesicles of multivesicular bodies (MVBs) [17]. MVBs play important roles in the sorting of substances into internal vesicles to ensure internal vesicle isolation from the cytoplasm and transport to the lysosomal lumen [17,18]. CHMP2B forms a long chain that spirals around the neck of the budding intracellular vesicles in MVBs [17,18]. Together with other components of ESCRT-III, CHMP2B constricts these intracellular vesicle necks immediately before the vesicles are severed from the membranes [17,18]. It is thus speculated that the dysfunction of CHMP2B results in moderate to severe neurological diseases and actually leads to the FTDALS spectrum [19,20].

During central nervous system (CNS) development, neuronal and glial cells undergo continuous and dynamic morphological differentiation [21,22,23,24]. In neuronal cells, morphogenesis includes a lot of stages, including the outgrowth and elongation of neurites, the navigation of neuronal processes, and the formation of synapses to form neuronal networks, together with interactions with glial cells [21,22,23,24,25,26]. Neurite outgrowth is the first step in establishing neuronal networks. However, all of the molecular mechanisms underlying various neuronal morphological differentiation stages, including neurite outgrowth, are still not completely understood. In neurological disorders, especially in neurodegenerative disorders, neuronal morphogenesis can be affected not only at an early stage but also at various other stages [27,28]. It is believed that abnormal morphogenesis in neuronal cells is critically associated with the onset or the specific phenotype of neurological disorders [27,28].

FTDALS type 7 (FTDALS7) is caused by various positions of mutations in CHMP2B [17,18,19,20]. Some mutations of CHMP2B predominantly have ALS symptoms, whereas other mutations predominantly have FTD symptoms. Among major mutations of CHMP2B, the Asp-148-to-Tyr mutation (p.Asp148Tyr [D148Y]) is preferentially responsible for FTD symptoms in FTDALS7 [17,18,19,20]. FTD is a sporadic or inherited disorder that affects the frontal and temporal lobes. While it is well known which mutation in CHMP2B is associated with the onset of FTD symptoms, it is unclear how the respective mutations in CHMP2B cause a cell biological effect to affect neuronal cell morphogenesis [17,18,19,20]. In the present study, we investigated whether CHMP2B with the D148Y mutation is abnormally localized in some organelles. We then asked whether cells expressing CHMP2B with the D148Y mutation blunt neuronal process elongation in rat primary cortical neurons and the N1E-115 cell line, which is a widely used model of neuronal differentiation [29,30,31,32]. Lastly, we explored if it is possible to restore cellular phenotypes by reducing organelle stress signals. These studies are expected to contribute to our understanding of the molecular and cellular pathological mechanism(s) underlying FTDALS7 and possible diseases affecting proteostasis.

## 2. Materials and Methods

### 2.1. Antibodies, siRNAs, and Plasmids

The key antibodies used and plasmids generated in this study are listed in Table 1.

### 2.2. Isolation and Culture of Primary Cortical Neuronal Cells

Primary cortical neuronal cells were isolated from rat cerebrum regions on embryonic days 16 to 17 and cultured as previously described [33,34]. Growing cultured cortical neuronal cells were detached by trypsinization once. In order to begin experiments to observe neuronal process elongation, neurons were once again attached to cultured dishes [3,34]. Rat cortical neuronal cells were transfected using the ScreenFect A transfection kit, according to the manufacturer’s instructions (Fujifilm, Tokyo, Japan). Neurons have approximately 5% transfection efficiency [33,34]. Under these conditions, attached cells incorporating trypan blue (Nacalai Tesque, Kyoto, Japan) were estimated to be less than 5% in each experiment.

### 2.3. Cell Culture and Differentiation

Green monkey kidney epithelial COS-7 cells and mouse neuronal N1E-115 (JCRB Cell Bank/Japan Health Sciences Foundation, Osaka, Japan) were cultured on cell and tissue culture dishes (Nunc, ThermoFisher Scientific, Waltham, MA, USA) in high-glucose Dulbecco’s modified Eagle medium (DMEM; Nacalai Tesque) containing 10% heat-inactivated fetal bovine serum (FBS) (ThermoFisher Scientific) and 100 unit/mL of penicillin–100 μg/mL streptomycin solution (ThermoFisher Scientific) in 5% CO_2_ at 37 °C. COS-7 and N1E-115 cells are known to have a high transfection efficiency and neuronal differentiation ability, respectively [29,30,31,32]. For differentiation experiments, ScreenFect A transfection kit-mediated cell lines stably expressing the wild-type (indicated as WT in the figure) *chmp2b* gene or the gene with the D148Y mutation (indicated as D148Y in the figure) were selected in the presence of 1 mg/mL of G418 (Nacalai Tesque), as previously described [29,30,31,32], and isolated as a single clone. To induce differentiation, N1E-115 cells were cultured in DMEM and 1% FBS containing 100 unit/mL of penicillin–100 μg/mL streptomycin solution in 5% CO_2_ at 37 °C in the presence or absence (dimethyl sulfoxide as the vehicle) of 10 μM of hesperetin for 2 days. Cells with processes more than two cell bodies in length were considered to be process-bearing cells (i.e., differentiated cells) [31,32]. Under these conditions, attached cells incorporating trypan blue were estimated to be less than 5% in each experiment [31,32].

### 2.4. Plasmid and siRNA Transfection

Cells were transiently transfected with plasmids and synthesized 21 mer small interfering (si)RNAs with dTdT using the ScreenFect A transfection kit and the ScreenFect siRNA transfection kit (Fujifilm), respectively, in accordance with the manufacturer’s instructions. The medium was replaced 4 h after transfection and was generally used for 48 to 72 h after transfection for cell biological and biochemical experiments. Under these conditions, attached cells incorporating trypan blue were estimated to be less than 5% in each experiment.

### 2.5. Denatured Polyacrylamide Electrophoresis and Immunoblotting

Cells were lysed in lysis buffer (50 mM HEPES-NaOH, pH 7.5, 150 mM NaCl, 3 mM MgCl_2_, 1 mM dithiothreitol, 1 mM phenylmethane sulfonylfluoride, 1 μg/mL leupeptin, 1 mM EDTA, 1 mM Na_3_VO_4_, 10 mM NaF, and 0.5% NP-40). For normal denatured conditions, cell lysates were denatured in sample buffers (Fujifilm), and samples were separated on a sodium dodecylsulfate polyacrylamide gel (Nacalai Tesque). The electrophoretically separated proteins were transferred to a polyvinylidene fluoride membrane (Fujifilm), blocked with Blocking One (Nacalai Tesque), and immunoblotted using primary antibodies, followed by peroxidase-enzyme-conjugated secondary antibodies. Peroxidase-reactive bands were captured using an image scanner (Canon, Tokyo, Japan) and scanned using CanoScan software (https://canon.jp/support/software/ (accessed on 1 July 2023)). The blots shown in the figures are representative of 3 blots. We performed some sets of experiments in immunoblotting studies and quantified other immunoreactive bands with one control’s immunoreactive band at 100% using the Image J software (https://imagej.nih.gov/ (accessed on 1 July 2023)).

### 2.6. Fluorescence Images

Cells on coverslips were fixed with 4% paraformaldehyde or 100% cold methanol (Nacalai Tesque) and blocked with Blocking One Histo (Nacalai Tesque). Slides were incubated with primary antibodies and then with Alexa Fluor fluorescent-conjugated secondary antibodies. The coverslips were mounted using the Vectashield kit (Vector Laboratories, Burlingame, CA, USA). The fluorescent images were collected and merged with the microscope systems FV1200 or FV3000 equipped with a laser-scanning Fluoview apparatus and software (both from Olympus, Tokyo, Japan (accessed on 10 January 2023)). The images in the figures are representative of 3 images and were analyzed using the Image J software (https://imagej.nih.gov/ (accessed on 1 July 2023)).

### 2.7. Statistical Analyses

Values are shown as means ± standard deviation (SD) of separate experiments. Intergroup comparisons were made using unpaired Student’s *t*-test in Excel (Microsoft, Redmond, WA, USA (accessed on 1 July 2023)). A one-way analysis of variance (ANOVA) was followed by Tukey’s multiple comparison test using Graph Pad Prism (GraphPad Software, San Diego, CA, USA (accessed on 1 July 2023)). Differences were considered statistically significant when *p* < 0.05.

### 2.8. Ethics Statement

Techniques using genetically modified cells and related techniques were performed in accordance with a protocol approved by the Tokyo University of Pharmacy and Life Sciences Gene and Animal Care Committee (Approval Nos. LS28-20 and LSR3-011).

## 3. Results

### 3.1. Cells Harboring CHMP2B with the D148Y Mutation form Aggregate-like Structures in the Golgi Body, Whereas Wild-Type CHMP2B Is Contained in MVB-like Structures

**Figure 1:**
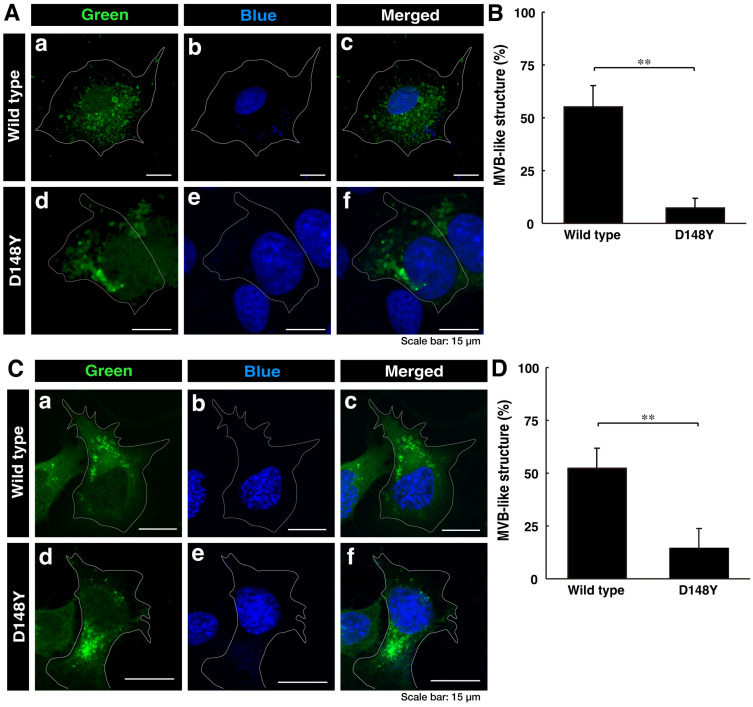
Wild-type CHMP2B is present in MVB-like structures, whereas CHMP2B with the D148Y mutation forms aggregate-like structures. (**A**) COS-7 cells were transiently transfected with the plasmid encoding wild-type CHMP2B tagged with EGFP (**a**–**c**) at its C-terminus or EGFP-tagged CHMP2B with the D148Y mutation (**d**–**f**). Successfully transfected cells with CHMP2B (green, (**a**,**d**)) were also stained with DAPI to detect nuclear positions (blue, (**b**,**e**)). Merged images are also depicted in (**c**,**f**). It is possible that the nuclei of COS-7 cells have some amplified chromosomes and/or nuclei arrested in the middle of cell division. (**B**) Cells with MVB-like structures were statistically depicted in the graph (** *p* < 0.01; n = 3 fields). (**C**) N1E-115 cells were transiently transfected with the plasmid encoding wild-type CHMP2B tagged with EGFP (**a**–**c**) at its C-terminus or EGFP-tagged CHMP2B with the D148Y mutation (**d**–**f**). Successfully transfected cells with CHMP2B (green, (**a**,**d**)) were also stained with DAPI to detect nuclear positions (blue, (**b**,**e**)). Merged images are also depicted in (**c**,**f**). (**D**) Cells with MVB-like structures are statistically depicted in the graph (** *p* < 0.01; n = 3 fields).

First, we investigated whether CHMP2B with the D148Y mutation is able to be localized in MVB-like structures or other intracellular structures. We transiently transfected the plasmid encoding CHMP2B protein with the D148Y mutation or wild-type CHMP2B protein into COS-7 cells. Since COS-7 cells have wide cytoplasmic regions, they are used to determine the localization of the gene product [32]. In confocal laser microscopy, wild-type CHMP2B was indeed localized in MVB-like structures within cells, whereas cells expressing CHMP2B with the D148Y mutation contained some aggregate-like structures (Figure 1A,B). Both transfected and nontransfected cells were stained with DAPI. Similar results were obtained in the case of the N1E-115 cell line as a neuronal cell model (Figure 1C,D).

To further examine whether CHMP2B with the D148Y mutation is localized in the organelle structure, we transfected the plasmid encoding CHMP2B with the D148Y mutation into N1E-115 cells and stained them with the respective antibodies against the endoplasmic reticulum (ER) antigen Lys-Asp-Glu-Leu (KDEL), Golgi body antigen 130 kDa Golgi body protein (GM130), and lysosome-specific antigen cathepsin D (Figure S1). Neither wild-type nor mutated CHMP2B was co-stained with the ER antigen in confocal laser microscopy. Wild-type CHMP2B was not co-stained with the Golgi body antigen, but mutated CHMP2B was co-stained with its antigen. In addition, neither wild-type nor mutated CHMP2B was significantly co-stained with the lysosome antigen, illustrating that mutated CHMP2B forms aggregate-like structures in both COS-7 cells and N1E-115 cells. Furthermore, mutated CHMP2B was found to be preferentially localized in the Golgi body.

**Figure 2:**
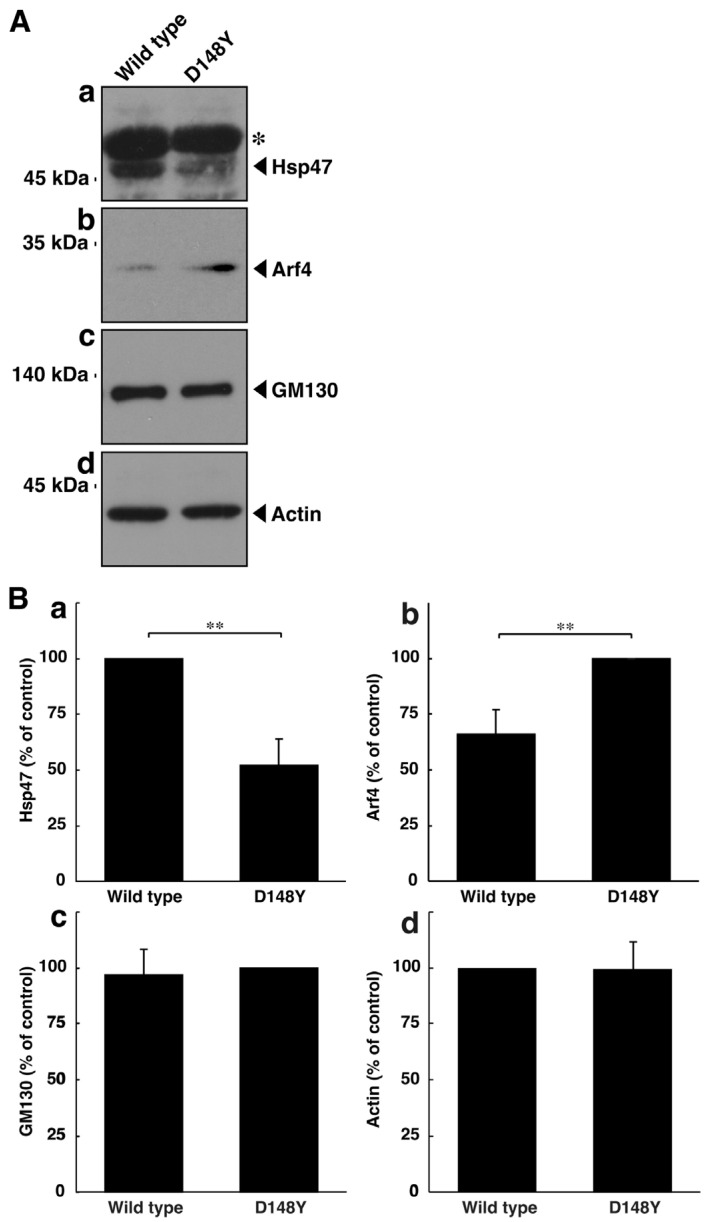
CHMP2B with the D148Y mutation alters the expression levels of molecules associated with Golgi stress. (**A**) N1E-115 cells were transfected with the plasmid encoding wild-type CHMP2B tagged with EGFP or EGFP-tagged CHMP2B with the D148Y mutation. The lysates of transfected cells were immunoblotted with the respective Golgi stress molecule antibodies against Hsp47 (**a**), Arf4 (**b**), GM130 (**c**), or control actin (**d**). In the Hsp47 blot, the asterisk (*) indicates probable non-specific immunoreactive bands. The protein bands slightly above 45 kDa correspond to the immunoreactive ones of Hsp47. (**B**) The immunoreactive bands were scanned, and each band was statistically analyzed ((**a**) Hsp47; (**b**) Arf4; (**c**) GM130; (**d**) actin) with the control band at 100% for (**a**,**d**) and with the band in the D148Y mutation at 100% for (**b**,**c**) (** *p* < 0.01; n = 3 blots).

Thus, we tested the possibility that mutated CHMP2B triggers organelle stress in the Golgi body. The molecular pathways associated with Golgi stress primarily involve those through (1) Hsp47 (also called serpin family H member 1 [SerpinH1]), (2) Arf4, and (3) structural proteins composing the Golgi body, such as GM130 [35,36,37,38,39,40]. Proteins such as Hsp47 and GM130 have protective effects on Golgi and cell homeostasis; in contrast, proteins such as Arf4 likely function as alarm-like molecules for Golgi stress [41,42,43,44,45,46]. N1E-115 cells harboring CHMP2B with the D148Y mutation exhibited decreased expression levels of Hsp47 (Figure 2A,B). On the other hand, cells harboring mutated CHMP2B increased the expression levels of Arf4. The expression levels of GM130 were comparable in cells harboring mutated CHMP2B and those harboring wild-type CHMP2B. These results suggest that mutated CHMP2B triggers Golgi stress through molecular pathways composed of Hsp47 and Arf4.

### 3.2. Cells Harboring CHMP2B with the D148Y Mutation Display Blunted Neuronal Morphological Changes

**Figure 3:**
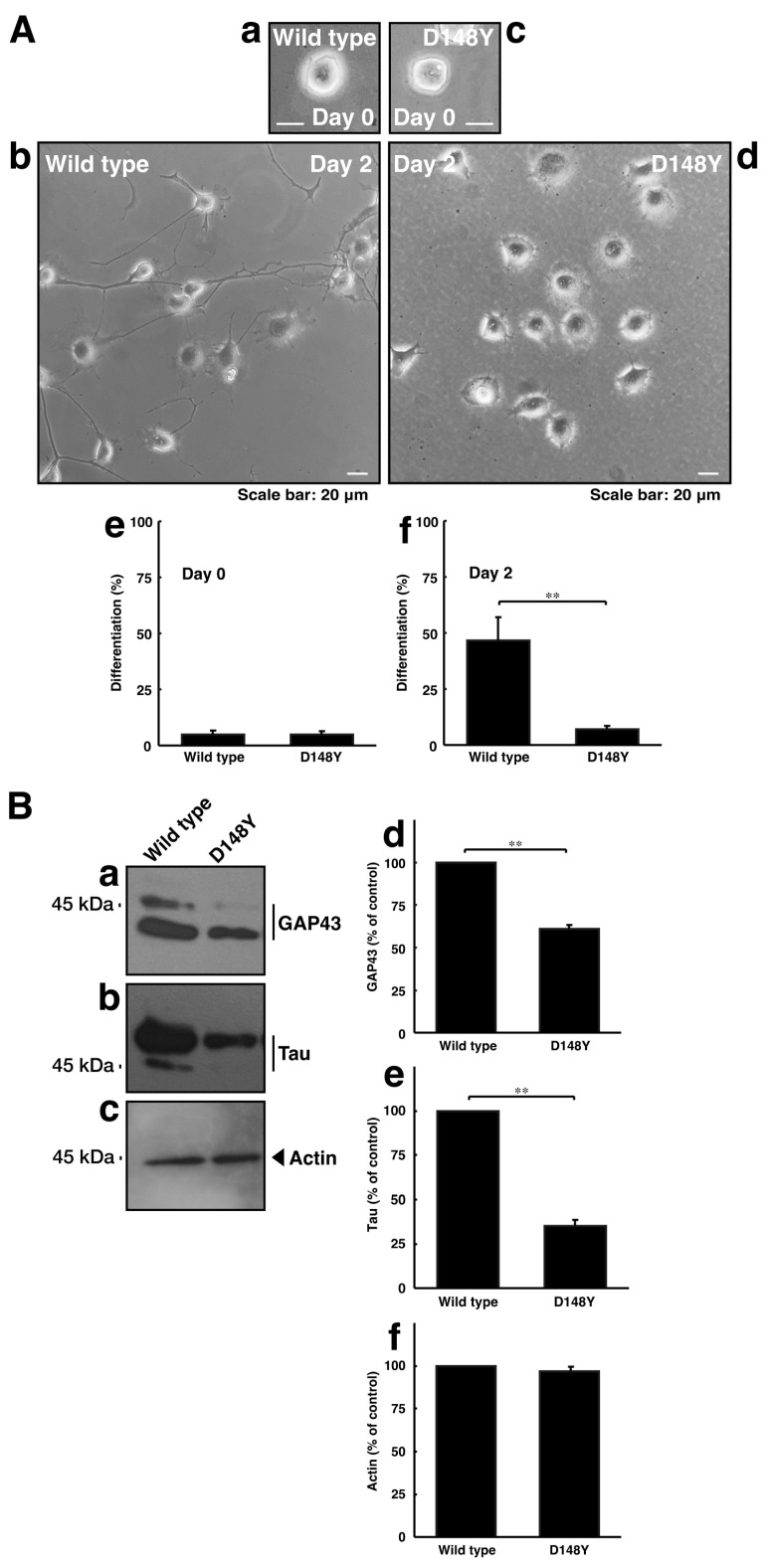
CHMP2B with the T148N mutation inhibits neuronal morphological differentiation with decreased expression levels of differentiation markers. (**A**) Cells harboring wild-type CHMP2B (**a**,**b**) or CHMP2B with the D148Y mutation (**c**,**d**) were allowed to differentiate for 0 (**a**,**c**) or 2 (**b**,**d**) days. Cells with processes with more than two cell body lengths were counted as differentiated cells at 0 (**e**) or 2 (**f**) days. Their cells are statistically depicted in the graph (** *p* < 0.01; n = 3 fields). (**B**) The lysates were immunoblotted with their respective differentiation marker antibodies against GAP43 (**a**), Tau (**b**), and actin (**c**). The immunoreactive bands were scanned, and each band was statistically analyzed with the control band (**d**–**f**) as 100% (** *p* < 0.01; n = 3 blots).

Since CHMP2B with the D148Y mutation failed to form MVB-like structures, we asked whether CHMP2B with the D148Y mutation affects neuronal morphological changes. The transfection of the plasmid encoding CHMP2B with the D148Y mutation resulted in shorter neuronal processes at day 3 in rat primary cortical neurons (Figure S2). In these microscopic images, the wild-type protein was localized in the cytoplasmic region around the nuclei. In contrast, mutated proteins also appeared to form small to large aggregates in primary neurons. Similar results on day 2 were observed in the case of N1E-115 cells (Figure 3A). These phenotypes were consistent with the results of decreased expression levels of neuronal marker proteins GAP43 and Tau in immunoblotting studies (Figure 3B), indicating that CHMP2B with the D148Y mutation affects the progression of neuronal morphological changes.

### 3.3. Neuronal Morphological Changes Can Be Recovered by Compensating Golgi Stress Signaling

**Figure 4:**
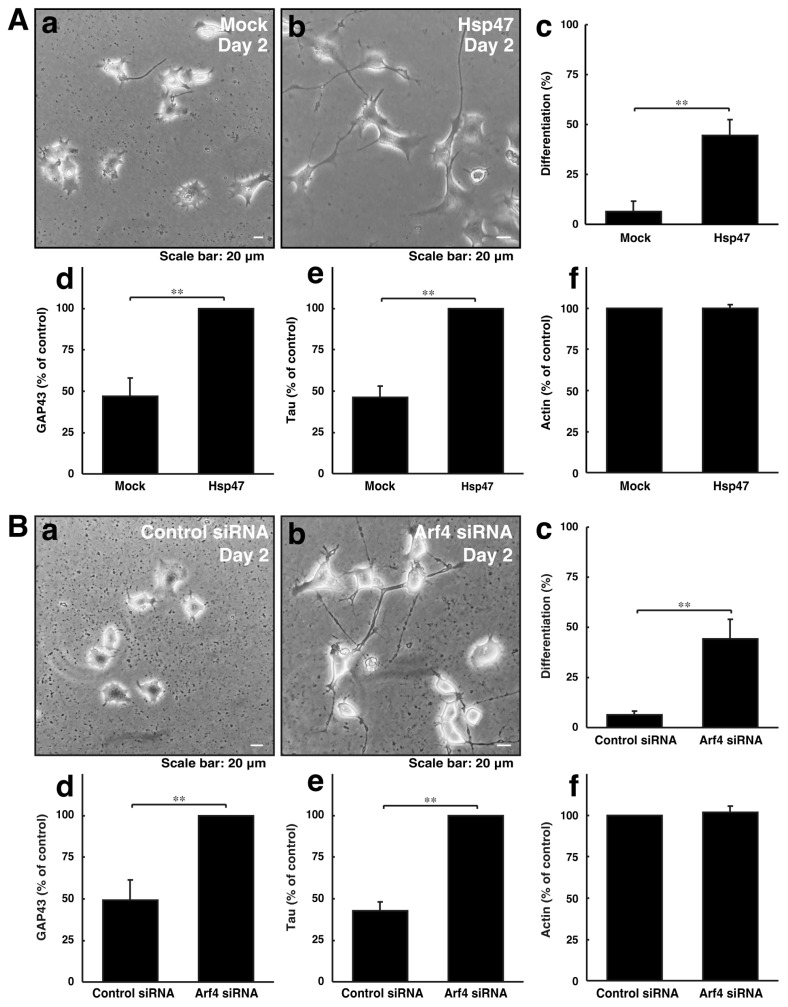
Modulating the expression levels of Golgi stress molecules recovers phenotypes of cells with CHMP2B with the T148N mutation with improved expression levels of differentiation markers. (**A**) Cells harboring CHMP2B with the D148Y mutation were transfected with mock (**a**) or Hsp47 (**b**) and allowed to differentiate for 2 days. Differentiated cells in cell images are statistically depicted (**c**) in the graph (** *p* < 0.01; n = 3 fields). The lysates were immunoblotted with an antibody against GAP43 (**d**), Tau (**e**), and actin (**f**). The immunoreactive bands were scanned, and each band was statistically analyzed with the control band at 100% for (**f**) and with the band in Hsp47 at 100% for (**d**,**e**) (** *p* < 0.01; n = 3 blots). (**B**) Cells harboring CHMP2B with the D148Y mutation were transfected with control siRNA (**a**) or Arf4 siRNA (**b**) and allowed to differentiate for 2 days. Differentiated cells are statistically depicted (**c**) in the graph (** *p* < 0.01; n = 3 fields). The lysates were immunoblotted with an antibody against GAP43 (**d**), Tau (**e**), and actin (**f**). The immunoreactive bands were scanned, and each band was statistically analyzed with the control band at 100% for (**f**) and with the band in the Arf4 siRNA at 100% for (**d**,**e**) (** *p* < 0.01; n = 3 blots).

We explored whether reducing Golgi stress signaling can recover cellular phenotypes induced by CHMP2B with the D148Y mutation. Since Hsp47 acts as a protective molecule for organelle stress in the Golgi body, we transfected the plasmid encoding Hsp47 into cells with CHMP2B with the D148Y mutation. In rat primary cortical neurons, Hsp47 recovered phenotypes with shorter neuronal processes (Figure S3). Arf4 acted as an alarm-like molecule responsible for Golgi stress, and its knockdown resulted in recovering phenotypes with shorter processes. Similarly, Hsp47 recovered phenotypes in N1E-115 cells harboring mutated CHMP2B (Figure 4A). In addition, the knockdown of Arf4 recovered phenotypes in cells harboring mutated CHMP2B (Figure 4B).

Although there is no consensus regarding all Golgi stress-signaling pathways [39,40,41,42,43,44], it is known that flavonoids such as hesperetin can reduce this stress through various Golgi stress-signaling pathways, including Hsp47, Arf4, and Golgi body structural proteins [35,36,37,38,39,40]. To further reconfirm that reducing Golgi stress can recover mutated CHMP2B-induced phenotypes in cells, we investigated whether hesperetin, serving as a positive control chemical, has a protective effect on cells harboring mutated CHMP2B. Treatment with hesperetin resulted in the recovery of morphological differentiation and marker expression in N1E-115 cells harboring mutated CHMP2B (Figure S4). This implies that mutated CHMP2B triggers Golgi stress, thereby impairing morphological differentiation in the neuronal cell line, and this effect can be alleviated by hesperetin (see Figures S5 and S6).

## 4. Discussion

FTDALS is an autosomal-dominant neurodegenerative disease that often develops in adults and shares the characteristics of FTD and ALS. This disease is characterized by the occurrence of one or both disease symptoms. This disorder is genetically and/or pathologically heterogeneous. There is also significant variation, even within families. For example, in the *c9orf72* gene responsible for FTDALS1, the gene products are proteins containing the differentially expressed in normal and neoplastic cells (DENN) domain acting as cytoplasmic guanine–nucleotide exchange factor (GEF) proteins for Rab family small GTPases, which participate in transporting intracellular vesicles in an integrated manner [1,2,3,4]. Most FTDALS1 patients have a specific nucleotide repeat structure in the gene. Patients have a younger age at onset and shorter survival times than patients with other types of FTDALSs. They also tend to have psychotic or hallucinatory tendencies. Patients with expanded repeats often develop dementia and have psychiatric disorders before its onset [1,2,3,4]. Many people with FTDALS2 also suffer from frontotemporal dementia but display many other aspects, including cerebellar ataxia, myopathy, motor neuron disease, and late-onset neurodegenerative diseases, similar to ALS. FTDALS2 is responsible for an amino acid replacement mutation of the mitochondrial coiled-coil-helix-coiled-coil-helix domain containing protein 10 (CHCHD10) [47,48]. As such, although FTDALSs involve a variety of symptoms and many responsible genes, they are considered to be classified as a single disease. Although it is believed that FTDALS-responsible genes can be classified into ones encoding functional proteins that control or affect proteostasis in cells, it sometimes appears that the functions of these gene products are not necessarily directly linked to proteostasis [13,14,15,16].

CHMP2B, as the product of the gene responsible for FTDALS7, is directly associated with the proteostasis pathway. CHMP2B is involved in the regulation of trafficking in intracellular vesicles from endosomes to lysosomes, which are essential in the endpoint of proteolysis [13,14,15,16]. CHMP2B with FTDALS7-associated mutations is likely related to the dysregulation of ESCRT machineries, ESCRT-0, ESCRT-I, ESCRT-II, and ESCRT-III, especially ESCRT-III. Through ESCRT machineries, ubiquitin-conjugated proteins are sequestered within the internal vesicles of MVBs. The sequestration results in the degradation of ubiquitin-conjugated proteins in the internal vesicles [17,18,19,20]. Also, the symptoms of FTDALS7 are unlike those of other FTDALSs, including FTDALS1 and FTDALS2 [17,18,19,20]. The symptoms appear to significantly differ depending on each patient. Patients with some mutations primarily display ALS, which manifests as muscle weakness in the upper and lower extremities, eye symptoms, and respiratory failure. Patients with other mutations display FTD, which manifests as changes in behavior and personality, memory loss, and a decline in cognitive function. There are also patients with both phenotypes; however, it remains to be elucidated whether and how each mutation in CHMP2B predisposes patients to ALS or FTD symptoms [17,18,19,20]. Further, the potential molecular mechanisms that preferentially cause ALS or FTD symptoms remain to be clarified. In the present study, we found that CHMP2B with the D148Y mutation is primarily accumulated in Golgi bodies but not in ERs. The mutant protein uniquely mediates some stress-signaling molecules in Golgi bodies. Further studies on the relationship between the respective mutants that cause FTD and/or ALS, which have been identified in FTDALS7, and types of Golgi stress signaling may provide us a hint on how the respective mutants preferentially cause FTD and/or ALS.

Accumulating reports have begun to classify the molecules responsible for Golgi stress into several pathways [35,36,37,38,39,40]. It is likely that Golgi stress is mediated by three major pathways through (1) an unidentified transcription factor and its possible downstream target, Hsp47, with a role in protecting cells; (2) the cAMP-responsive element binding protein 3 (CREB3) transcription factor and its downstream target, Arf4; and (3) transcription factor E3 (TFE3) and downstream targets such as the structural proteins composing the Golgi body, for example, GM130 [35,36,37,38,39,40]. It is believed that Hsp47 often composes an anti-apoptotic pathway in cell types such as fibroblasts [41,42], whereas signaling through CREB3 composes a pro-apoptotic, pre-apoptotic, and/or apoptotic pathway [43,44]. In addition, CREB3, acting through Arf4, can be linked to caspase-2 as an apoptotic protease family member [43,44]. Further, the TFE3 pathway likely provides a cross-talk platform for Golgi stress-signaling molecules being discovered one after another [45,46]. The Golgi stress pathway is thought to mediate the effects of some specific stresses within mammalian cells. Like the unfolded protein response (UPR) in ERs, part of these pathways is related to arresting the cell cycle, at least in fibroblasts [27]; however, the roles of Golgi stress responses in many other types of cells remain unclear [35,36,37,38,39,40].

Based on the above concept, we can discuss our results for each of the three major molecular pathways of Golgi stress responses. First, the expression levels of Hsp47 are specifically decreased in cells harboring CHMP2B with the D148Y mutation. Hsp47 is generally identified as an ER chaperone, but Hsp47 is actually localized in Golgi bodies, playing key roles in protecting Golgi bodies from various identified or unidentified stresses [41,42]. It is conceivable that the exogenous expression of Hsp47 can promote process elongation by protecting cells harboring CHMP2B with the D148Y mutation in neuronal-type cells, although it remains unclear how Hsp47 protects cells to promote morphological changes. Second, expression levels of Arf4 are increased in cells harboring CHMP2B with the D148Y mutation compared with cells harboring the wild type. It is likely that a possible CREB3 pathway responds to Golgi stress induced by mutated CHMP2B proteins, acting as one of the alarm molecules in Golgi stress [43,44]. The knockdown of Arf4 results in promoted process elongation in cells. Third, expression levels of GM130, the enriched Golgi body structural protein, are unlikely to affect cells harboring CHMP2B with the D148Y mutation. TFE3 is an essential transcription factor controlling the genes that encode Golgi body structural proteins, the intracellular vesicle transporting molecules, and Golgi-resident enzymes that mediate glycosylation [45,46]. Since the expression levels of GM130 appear to be unchangeable in cells harboring CHMP2B with the D148Y mutation, the TFE3-mediated pathway may have been able to cope with the Golgi stress induced by the mutated CHMP2B protein. These results do not correspond to expression profiles of Golgi stress-responsible molecules in cells expressing the mutant protein that preferentially causes ALS [5,6,7,8]. It is possible that, at least at the molecular and cellular levels, differences in molecular expression and/or activities in stress sensing in Golgi bodies may provide a hint to explain the relationship between mutated positions in CHMP2B proteins and the development of FTD and/or ALS.

Unlike other heat-shock protein family members, Hsp47 structurally belongs to a member of the serpin superfamily among intracellular inhibitors for serine proteinases. However, Hsp47 has few to no activities as a serine protease inhibitor [49,50,51,52]. It is often present in the ER lumen through its C-terminal RDEL sequence, associates with procollagen of high-molecular-weight protein, and transports it to the cis-Golgi or ER-Golgi intermediate compartment (ERGIC). Hsp47 recognizes GXR (X indicates any amino acid) repeats on procollagen to suppress the formation of unfolding and/or the aggregation of procollagen [49,50,51,52]. It is, therefore, thought that Hsp47 exists in Golgi bodies and in ERs [49,50,51,52]. In addition, since Hsp47, at least virtually, binds to many types of proteins (see the BioGrid website, https://thebiogrid.org (accessed on 1 September 2023)), it is presumed that, like other heat-shock protein family members, Hsp47 has a role in escorting other proteins in Golgi and ER lumens [49,50,51,52]. This reminds us that Grp78 (also called BiP or heat-shock protein family A (Hsp70) member 5 [HSPA5]) protectively binds to unfolded and/or aggregated proteins in the ER lumen, helping activate the ER stress sensor receptor [27]. The expression levels of Grp78 itself are increased by accepting weak or moderate stress signaling, and Grp78 participates in forming positive feedback [27]. Herein, we found that the expression levels of Hsp47 proteins are specifically decreased in cells expressing CHMP2B with the D148Y mutation, which aberrantly affects cell morphological changes. Conversely, the transfection of Hsp47 triggers morphological changes. It is conceivable that Hsp47 has some protective effects on unfolded and/or aggregated proteins in the Golgi lumen.

Arf4 is a small GTPase family member and is thought to control intracellular vesicle trafficking with other Arf family small GTP-binding proteins [53,54,55,56]. Arf family members are composed of class I (Arf1, Arf2, and/or Arf3), class II (Arf4 and Arf5), and class III (Arf6) proteins [53,54,55,56]. While class I and III proteins are well characterized in terms of their roles in intracellular vesicle trafficking, little is known about the role of class II proteins [53,54,55,56]. It is clear that Arf1 and Ar4 are localized on the outside surface of Golgi bodies, but it remains to be determined how Arf4 is responsible for transport to organelles [53,54,55,56]. It is likely that Arf4 is involved in the regulation of transport around Golgi bodies strictly by monitoring them [53,54]. In fact, the knockdown of Arf4 increases resistance against pathogens, including *Chlamydia trachomatis* and *Shigella flexneri*, mimicking the effects of brefeldin A (BFA), blocking protein transport around Golgi bodies [55,56]. We observe that the expression levels of Arf4 proteins are specifically increased in cells expressing CHMP2B with the D148Y mutation concomitantly with morphological changes; in contrast, the knockdown of Arf4 recovers cell morphological changes. Arf4 knockdown may be able to reduce some stresses on Golgi bodies.

## 5. Conclusions 

This study marks the first description of the impact of CHMP2B with the D148Y mutation on the expression levels of Golgi stress regulators, Hsp47 and Arf4, and their influence on neuronal cell morphological differentiation. Transfection or knockdown of Hsp47 or Arf4 has been shown to restore their progression, indicating an association between aberrant expression levels of Golgi stress regulators and defects in neuronal differentiation.

Further studies will increase our understanding not only of the detailed molecular mechanisms by which the expression levels of Golgi stress regulators are changed in neuronal cells but also of how these regulators affect neuronal cell morphological changes in neuronal cells. Additional studies will help clarify the bona fide relationship between Golgi stress signaling and various neurological diseases, both in vitro and in vivo. Such a series of studies could lead to the development of drugs targeting the molecular mechanisms implicated in CHMP2B-related diseases and other FTDALSs at the molecular and cellular levels.

## Figures and Tables

**Table 1:** Key antibodies, siRNAs, and plasmids used in this study.

Reagent or Material	Company or Source	Cat. No.	Lot No.	Concentration Used
Antibody				
Anti-heat-shock protein 47 (Hsp47)	Santa Cruz Biotechnology (Santa Cruz, CA, USA)	sc-5293	I2118	Immunoblotting (IB), 1:200
Anti-Arf4	Proteintech Japan (Tokyo, Japan)	11673-1-AP	00048284	IB, 1:1000
Anti-Actin	MBL (Tokyo, Japan)	M177-3	007	IB, 1:1000
Anti-Growth associated protein 43 (GAP43)	Santa Cruz Biotechnology	sc-17790	J0920	IB, 1:8500
Anti-Tau	Santa Cruz Biotechnology	sc-21796	H2923	IB, 1:500
Anti-Lys-Asp-Glu-Leu (KDEL)	MBL	M181-3	004	Immunofluorescence (IF), 1:200
Anti-Golgi matrix protein of 130 kDa (GM130)	BD (Franklin Lakes, NJ, USA)	610823	8352796	IB, 1:500 and IF, 1:200
Anti-Cathepsin D	Abcam (Cambridge, UK)	ab75852	GR260148-33	IF, 1:200
Anti-IgG (H+L chain) (Rabbit) pAb-HRP	MBL	458	353	IB, 1:5000
Anti-IgG (H+L chain) (Mouse) pAb-HRP	MBL	330	365	IB, 1:5000
Alexa Fluor TM 594 goat anti-mouse IgG (H+L)	ThermoFisher Scientific (Waltham, MA, USA)	A11005	226-8383	IF, 1:500
Alexa Fluor TM 594 goat anti-rabbit IgG (H+L)	ThermoFisher Scientific	A11012	201-8240	IF, 1:500
siRNA and recombinant DNA				
5′-GACGACAAUUCUGUAUAAA-dTdT-3′ and 5′-UUUAUACAGAAUUGUCGUC-dTdT-3′	siRNA duplex for Arf4 (generated in this study)	Not applicable		50 nM
5′-GCCAUUCUAUCCUCUAGAG-dTdT-3′ and 5′-CUCUAGAGGAUAGAAUGGC-dTdT-3′	siRNA duplex for *P. pyralis* luciferase (generated in this study)	Not applicable		50 nM
pcDNA3.1(+)-human Hsp47	GenScript (generated in this study)	Not applicable		1.25 mg of DNA per 6 cm dish
pEGFP-N3-human CHMP2B	Generated in this study	Not applicable		1.25 mg of DNA per 6 cm dish
pEGFP-N3-human CHMP2B with the D148Y mutation	Generated in this study	Not applicable		1.25 mg of DNA per 6 cm dish

## Data Availability

The original data are restricted from being disclosed for our further studies. All of the data not restricted are contained within the article or Supplementary Materials. However, limited disclosure may be upon request with an appropriate explanation from the corresponding author.

## Supplementary Materials

The following supporting information can be downloaded at https://www.mdpi.com/article/10.3390/cimb46020090/s1. Figure S1. CHMP2B with the D148Y mutation forms aggregate-like structures in the Golgi body; Figure S2. CHMP2B with the D148Y mutation inhibits axon elongation in cortical neurons; Figure S3. Modulating the expression levels of Golgi stress molecules recovers phenotypes of cortical neurons with CHMP2B with the D148Y mutation; Figure S4. Hesperetin, a chemical with protective effects on the Golgi body, recovers phenotypes of cells with CHMP2B with the D148Y mutation; Figure S5. Scanned original size gels (full size gels) for Figure 2; Figure S6. Scanned original size gels (full size gels) for Figure 3.
