# Supplementary material for: Modulating Golgi Stress Signaling Ameliorates Cell Morphological Phenotypes Induced by CHMP2B with Frontotemporal Dementia-Associated p.Asp148Tyr
Source: Curr Issues Mol Biol. 2024 Feb 5;46(2):1398–412. doi: 10.3390/cimb46020090 (PMC10888485; doi:10.3390/cimb46020090)
Supplement: Supplementary file 1 [file cimb-46-00090-s001.zip › 01 Supplematnal legends.pdf]

**Supplemental figure legends**

**Figure S1. CHMP2B with the D148Y mutation forms aggregate-like structures in the Golgi body.** (A) N1E-115 cells were transfected with the plasmid encoding GFP-tagged wild type CHMP2B (green) or GFP-tagged CHMP2B (green) with the D148Y mutation. Transfected cells were stained with an antibody against ER-specific antigen KDEL (red), Golgi body-specific antigen GM130 (red), or lysosome-specific antigen cathepsin D (red). (B) Scan plots were performed along the white lines in the direction of the arrows in the green, red, and merged images. Graphs showing green and red fluorescence intensity (F.I.; arbitrary units) along the lines in the direction of the arrows were depicted in the bottom panels.

**Figure S2. CHMP2B with the D148Y mutation inhibits axon elongation in cortical neurons.** (A) Rat cortical neurons were detached by trypsinization, reattached on culture dishes, and transfected with the plasmid encoding GFP-tagged wild type CHMP2B (green, a and b) or GFP-tagged CHMP2B (green, c and d) with the D148Y mutation. Their cells were allowed to elongate axons for 0 (a, c) or 3 (b, d) days. (B) Cells with processes measuring more than two cell body lengths were counted as differentiated cells for 0 (a) or 3 (b) days and statistically depicted in the graph (** $p<0.01$; n = 3 culture dishes).

**Figure S3. Modulating the expression levels of Golgi stress molecules recovers phenotypes of cortical neurons with CHMP2B with the D148Y mutation.** (A) Rat cortical neurons were detached by trypsinization, reattached on culture dishes, and transfected with the plasmid encoding GFP-tagged CHMP2B with the D148Y mutation together with (c, d) or without (a, b) the plasmid expressing Hsp47 for 0 (a, c, e) or 3 (b, d, f) days. Cells with processes measuring more than two cell body lengths were counted as differentiated cells (** $p<0.01$; n = 3 culture dishes). (B) Rat cortical neurons were detached by trypsinization, reattached on culture dishes, and transfected with the plasmid encoding GFP-tagged CHMP2B with the D148Y mutation together with control siRNA (a, b) or Arf4 siRNA (c, d) for 0 (a, c, e) or 3 (b, d, f) days. Cells with processes measuring more than two cell body lengths were counted as differentiated cells (** $p<0.01$; n = 3 culture dishes).

**Figure S4. Hesperetin, a chemical with protective effects on the Golgi body, recovers phenotypes of cells with CHMP2B with the D148Y mutation.** (A)N1E-115 cells harboring CHMP2B with the D148Y mutation were allowed to differentiate in the absence (a, vehicle control) or the presence (b) of 10 μM of hesperetin for 2 days. Differentiated cells in cell images were statistically depicted (c) in the graph (** $p<0.01$; n = 3 fields). The lysates were immunoblotted with an antibody against GAP43 (d), Tau (e), and actin (f). The immunoreactive bands were scanned and each band was statistically analyzed with the control band (f) as 100% or with the band in the presence of hesperetin (d, e) as 100% (** $p<0.01$; n = 3 blots).

**Figure S5. Scanned original size gels (full size gels) for Figure 2.**

**Figure S6. Scanned original size gels (full size gels) for Figure 3.**
