# Supplementary figures and images for: Modulating Golgi Stress Signaling Ameliorates Cell Morphological Phenotypes Induced by CHMP2B with Frontotemporal Dementia-Associated p.Asp148Tyr
Source: Curr Issues Mol Biol. 2024 Feb 5;46(2):1398–412. doi: 10.3390/cimb46020090 (PMC10888485; doi:10.3390/cimb46020090)

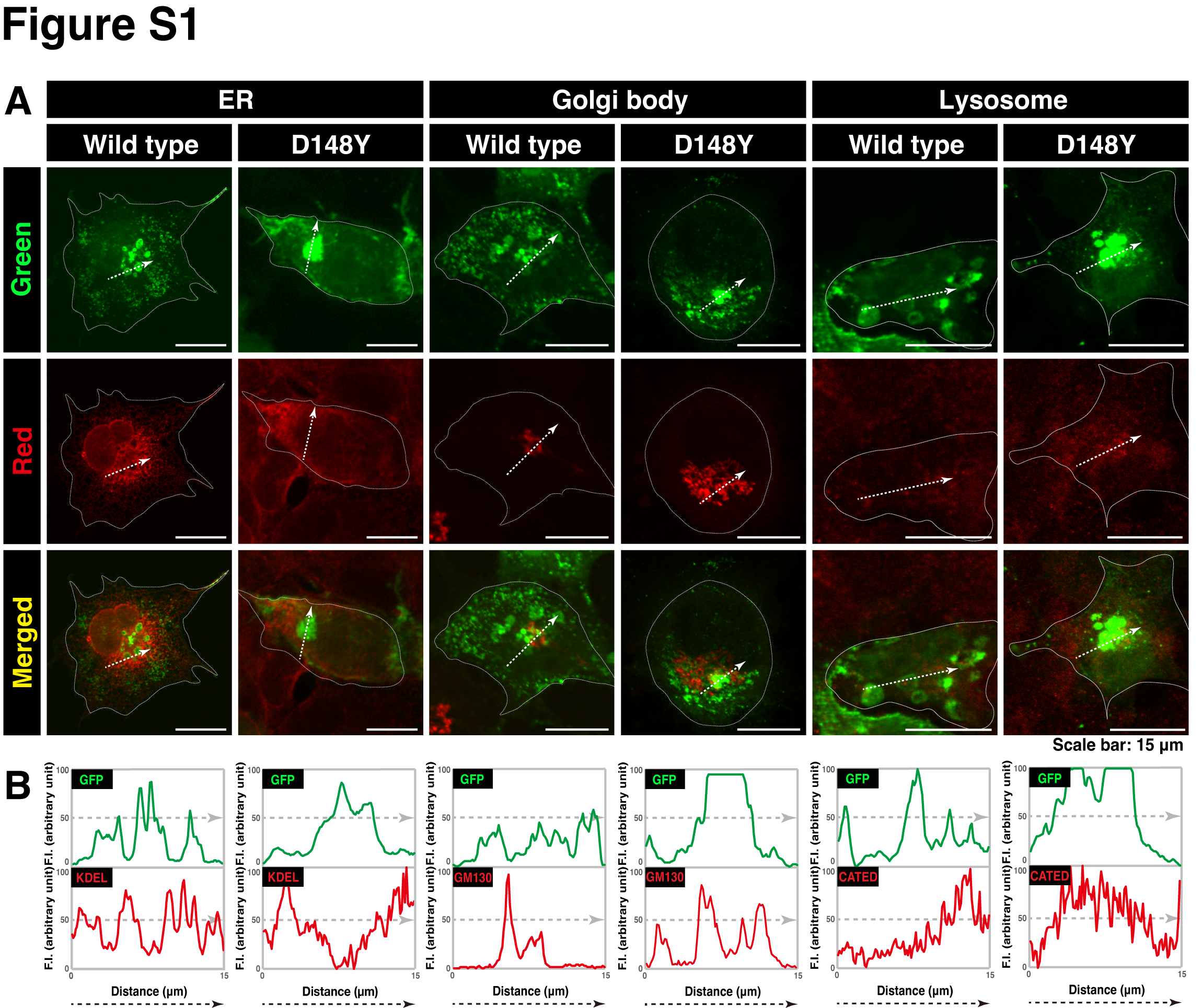

Supplement: Supplementary file 1 [file cimb-46-00090-s001.zip › Figure S1.tif]

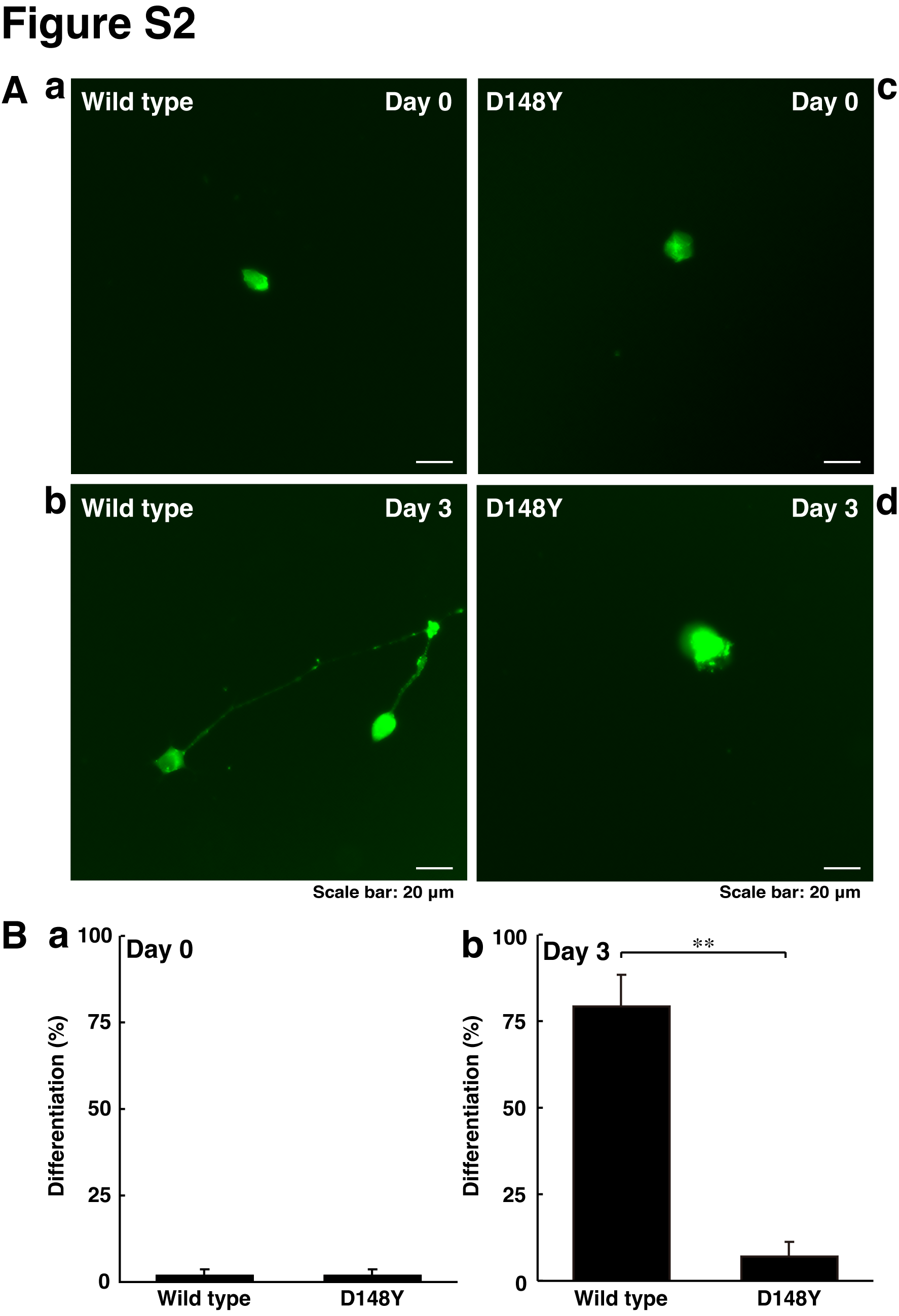

Supplement: Supplementary file 1 [file cimb-46-00090-s001.zip › Figure S2 Cortical.tif]

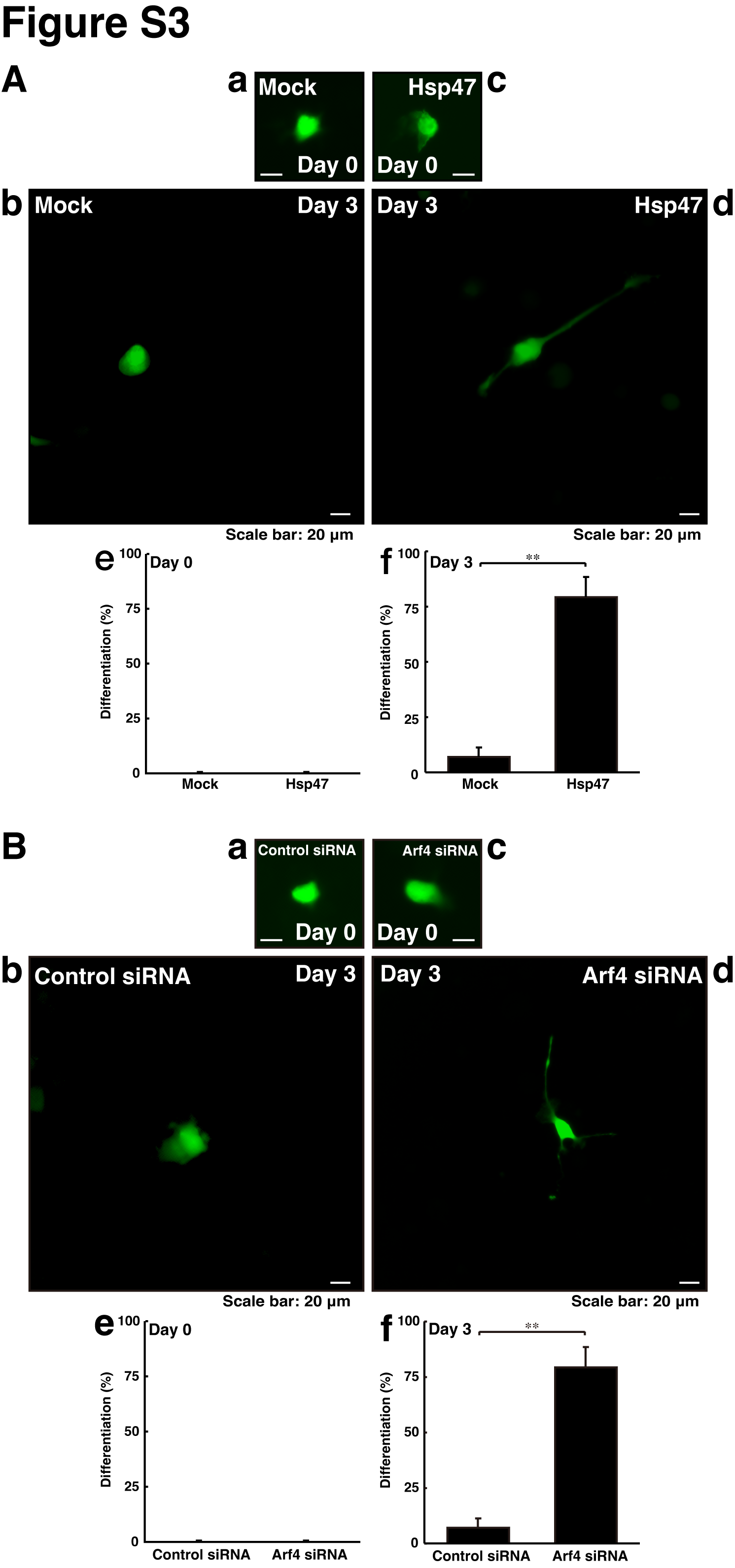

Supplement: Supplementary file 1 [file cimb-46-00090-s001.zip › Figure S3 Cortical.tif]

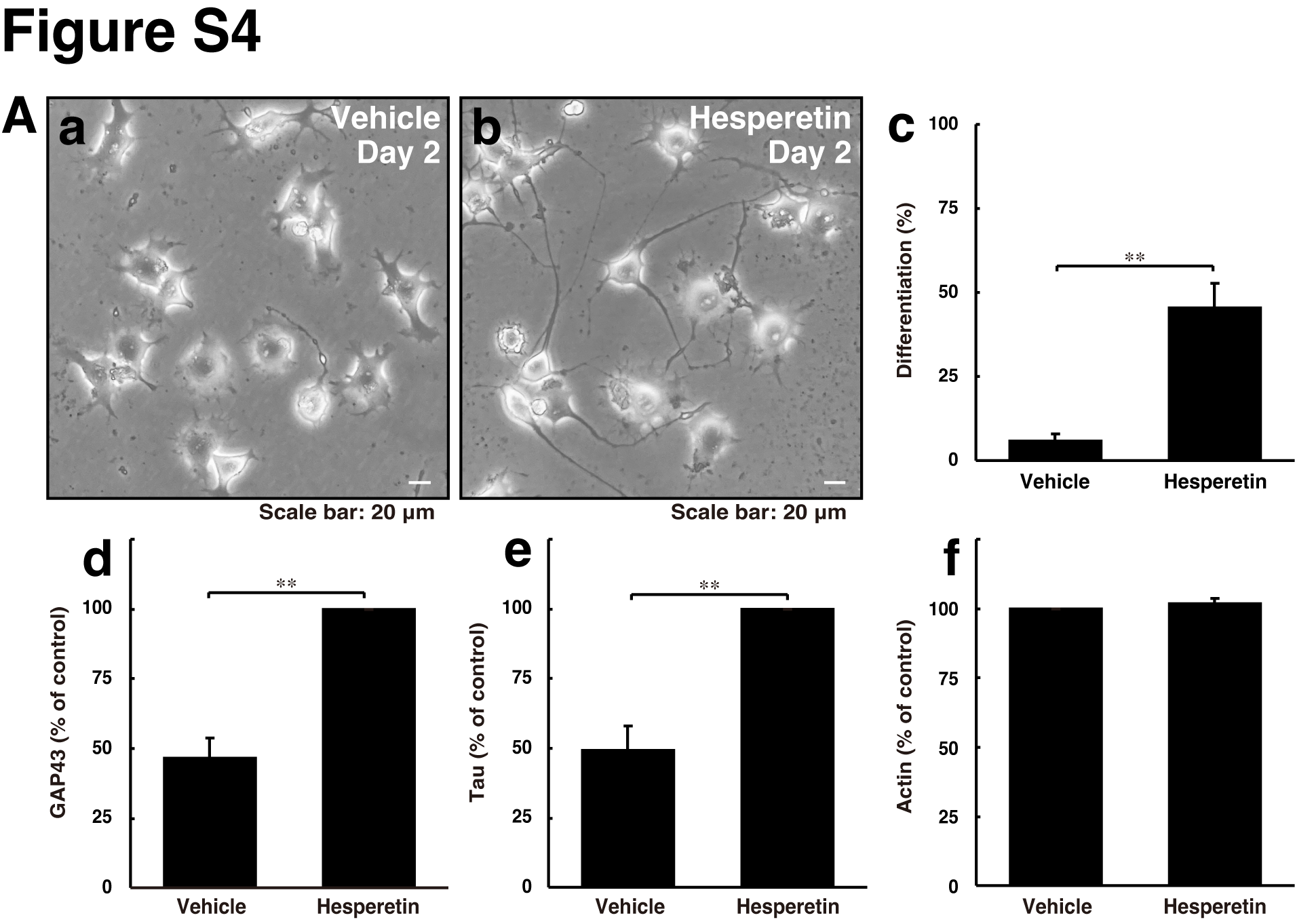

Supplement: Supplementary file 1 [file cimb-46-00090-s001.zip › Figure S4.tif]

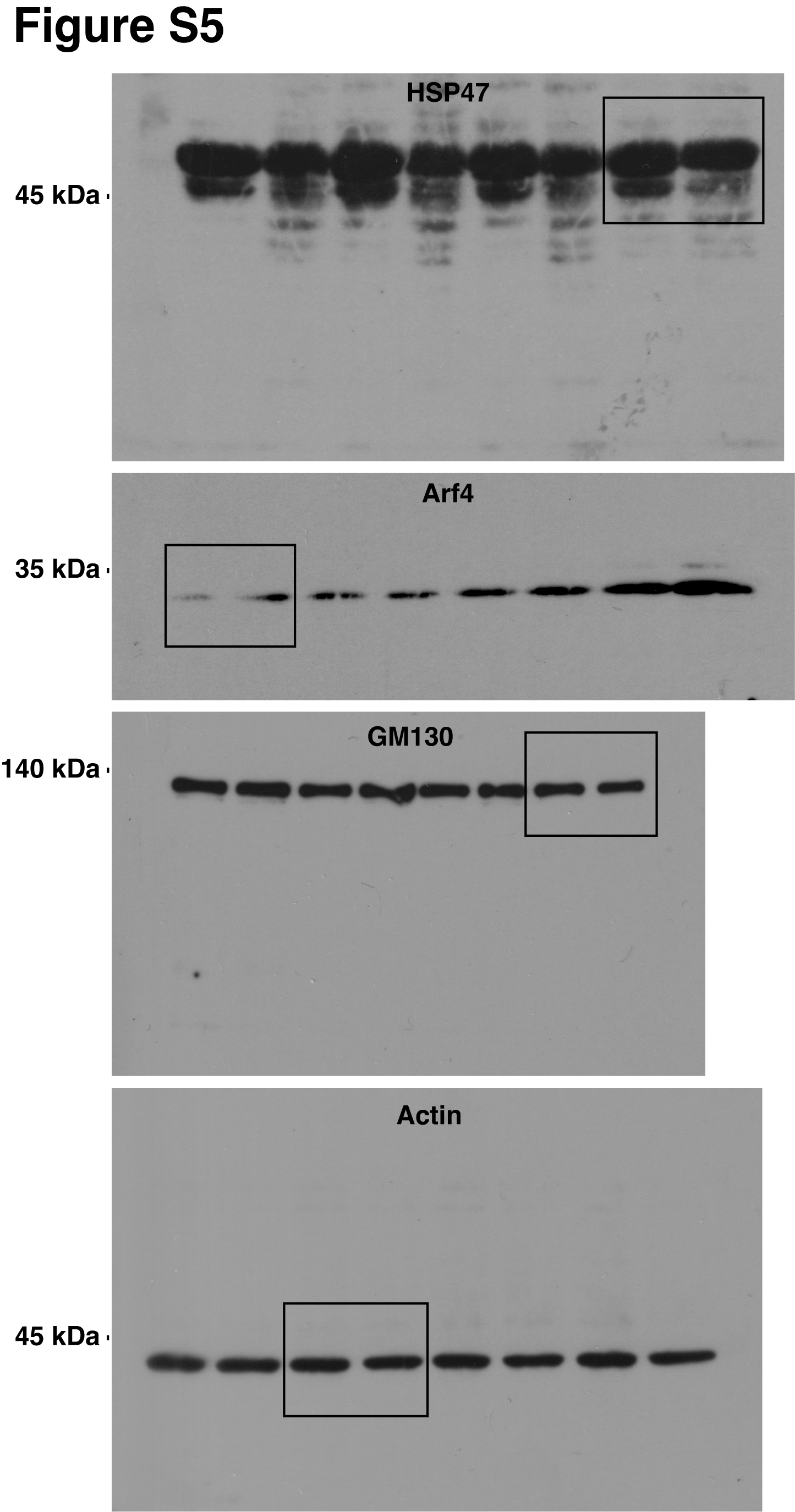

Supplement: Supplementary file 1 [file cimb-46-00090-s001.zip › Figure S5 for Figure 2.tif]

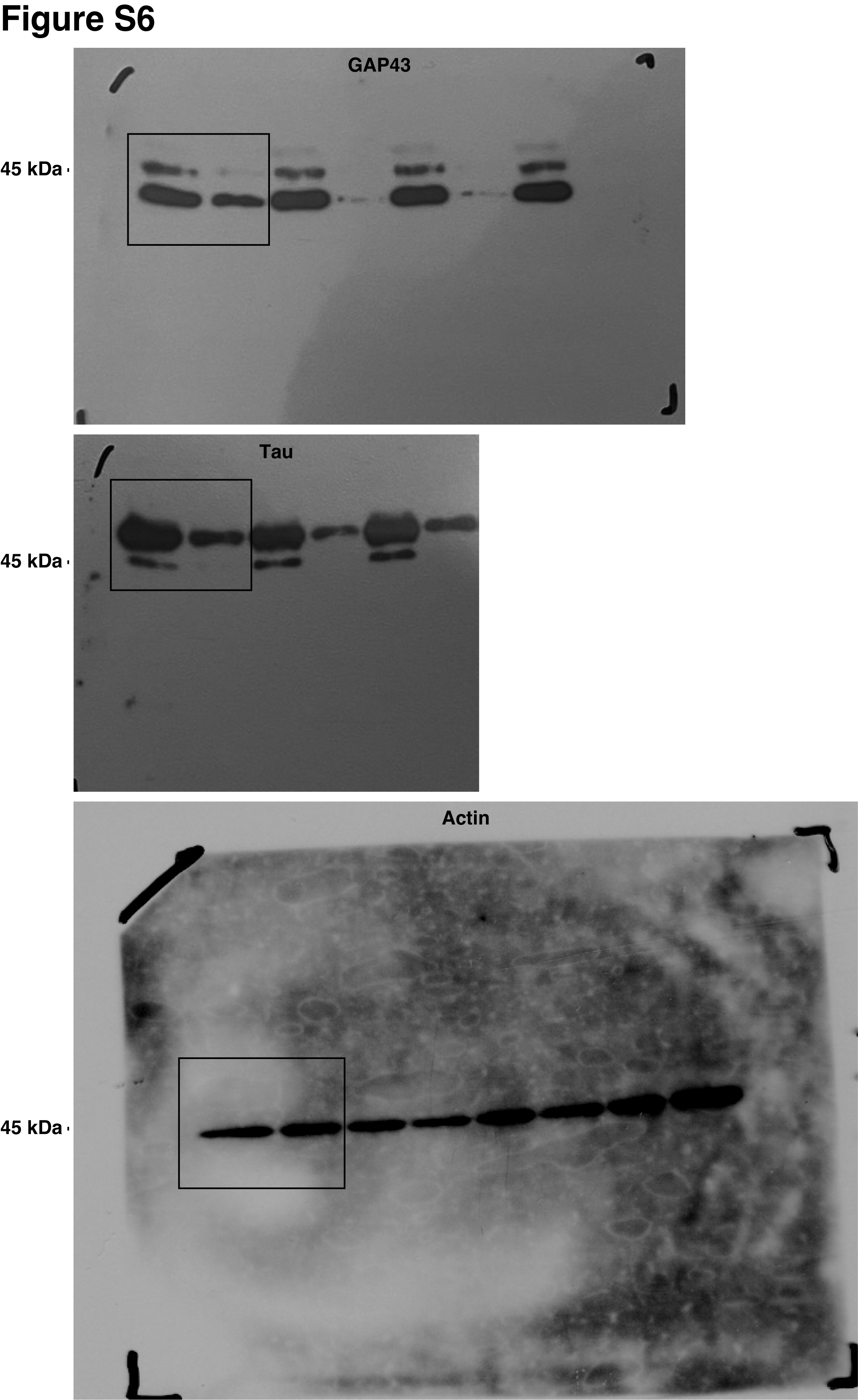

Supplement: Supplementary file 1 [file cimb-46-00090-s001.zip › Figure S6 for Figure 3.tif]
